# Vacuum Deposited Perovskites with a Controllable Crystal Orientation

**DOI:** 10.1021/acs.jpclett.3c01920

**Published:** 2023-09-25

**Authors:** Jin Yan, Lena Sophie Stickel, Lennart van den Hengel, Haoxu Wang, Prasaanth Ravi Anusuyadevi, Agnieszka Kooijman, Xiaohui Liu, Bahiya Ibrahim, Arjan Mol, Peyman Taheri, Luana Mazzarella, Olindo Isabella, Tom J. Savenije

**Affiliations:** † PVMD Group, Delft University of Technology, Mekelweg 4, 2628 CD Delft, The Netherlands; ‡ Department of ChemE, Delft University of Technology, Van der Maasweg 9, 2629 HZ Delft, The Netherlands; § Georg-August-University Göttingen, Göttingen 37077, Germany; ∥ Department of Materials Science and Engineering, Delft University of Technology, 2628 CD Delft, The Netherlands

## Abstract

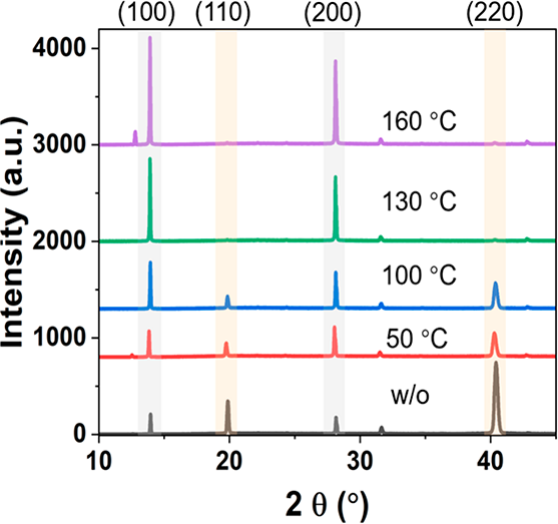

The preferential orientation of the perovskite (PVK) is typically accomplished by manipulation of the mixed cation/halide composition of the solution used for wet processing. However, for PVKs grown by thermal evaporation, this has been rarely addressed. It is unclear how variation in crystal orientation affects the optoelectronic properties of thermally evaporated films, including the charge carrier mobility, lifetime, and trap densities. In this study, we use different intermediate annealing temperatures *T*_inter_ between two sequential evaporation cycles to control the Cs_0.15_FA_0.85_PbI_2.85_Br_0.15_ orientation of the final PVK layer. XRD and 2D-XRD measurements reveal that when using no intermediate annealing primarily the (110) orientation is obtained, while when using *T*_inter_ = 100 °C a nearly isotropic orientation is found. Most interestingly for *T*_inter_ > 130 °C a highly oriented PVK (100) is formed. We found that although bulk electronic properties like photoconductivity are independent of the preferential orientation, surface related properties differ substantially. The highly oriented PVK (100) exhibits improved photoluminescence in terms of yield and lifetime. In addition, high spatial resolution mappings of the contact potential difference (CPD) as measured by KPFM for the highly oriented PVK show a more homogeneous surface potential distribution than those of the nonoriented PVK. These observations suggest that a highly oriented growth of thermally evaporated PVK is preferred to improve the charge extraction at the device level.

Metal halide perovskite (PVK) solar cells (PSCs) have attracted an extensive amount of attention due to the rapid enhancement of the power conversion efficiency (PCE) reaching 25% within a decade.^1^ The most explored approach to deposit high-quality PVK films is wet chemical processing including spin-coating, which has demonstrated excellent performing devices with relatively small areas of about 0.1–1 cm^2^.^2^ Various strategies have been reported to improve the PCE of spin-coated PSCs, including structure design,^3^ interface modification,^4^ and composition replacement for each functional layer.^5^ Among them, controlling the growth of PVK crystals is critical to obtain high-quality absorber materials that exhibit high absorption coefficients, high charge carrier mobilities, and long lifetimes.^6,7^ To date, several main techniques are commonly applied to control the PVK growth, such as additive engineering,^8^ solvent engineering,^9^ and gradient annealing.^10^ The main goal of these approaches is to slow down solvent evaporation to enhance the crystal grain size.^11^ Moreover, various groups have reported highly selective growth using template modulated PVK growth,^12,13^ or composition engineering, such as MA/FA mixed cations in precursor solution in ref (14). In these works, preferential crystal growth has been claimed to suppress nonradiative recombination and to improve the free carrier lifetime in spin-coated PVK layers.^14,15^ Therefore, manipulation of the PVK preferential crystal orientation is an interesting and effective method to improve the PCE of PSCs.^16,17^

Unlike spin coating, thermal evaporation of PVKs does not require the use of harmful solvents and is compatible with textured substrates.^18^ Besides, it has very good potential toward upscaling due to the exact precursor control^19^ and uniform deposition.^20^ Recently, Li et al.^21^ prepared minimodules based on thermally evaporated PVKs, with a champion PCE above 18% with an active area of 22 cm^2^. However, manipulating the preferential orientation of the PVK crystal growth in layers prepared by thermal evaporation has hardly been addressed.^22,23^ To date, only two papers have been reported on the control of the preferential growth of thermally evaporated PVKs. Abzieher et al.^24^ investigated the effects of substrate material on the orientation of methylammonium lead iodide (MAPbI_3_) crystals and identified few organic hole transport materials as ideal candidates for the fabrication of efficient fully evaporated PSCs.^24^ Similarly, Klipfel et al.^25^ highlighted the importance of the underlying material selection. Furthermore, both studies stressed that the deposition rate of coevaporated lead(II) iodide (PbI_2_) and methylammonium iodide (MAI) can achieve fine-tuning of preferred crystal orientation.^25^ However, these studies mainly focused on the influence of substrate material on MAPbI_3_ orientation growth and discussed optoelectrical properties at the device level. A systematic study of the influence of the preferential orientation on charge carrier mobility, lifetime, and trap densities is lacking.^26^ Besides, no strategies to manipulate the crystal orientation of thermally evaporated Cs_*x*_FA_1–*x*_PbI_3–*y*_Br_*y*_ have been reported. Therefore, it is important to manipulate the crystal orientation of thermally evaporated Cs_*x*_FA_1–*x*_PbI_3–*y*_Br_*y*_ and investigate the effects of the crystal orientation on its optoelectronic properties.

In this work, PVK films are fabricated using sequential deposition comprising two cycles, each consisting of three thick precursor layers, i.e., PbI_2_, formamidinium iodide (FAI), and cesium bromide (CsBr).^27^ By applying different intermediate annealing temperatures between the first and second cycles, we can fabricate 450 nm thick Cs_0.15_FA_0.85_PbI_2.85_Br_0.15_ films with a different preferable crystal orientation, as demonstrated by both X-ray diffraction (XRD) and two-dimensional XRD (2D-XRD). In addition, we explain the underlying mechanism of the controllable crystal orientation by XRD. Furthermore, we investigate how the PVK crystal orientation influences the optoelectronic properties in the bulk and at the surface of the film by performing photoluminescence (PL) measurements, time-resolved microwave conductivity (TRMC) measurements, and Kelvin probe force microscopy (KPFM). Our findings suggest that the preferential growth along (100) shows less variation in the surface potential/lower trap densities on the film surface compared with the (110)/(100) mixed one. On the other hand, the variation of crystal orientation shows no effects on the bulk optoelectrical properties for the thermally evaporated Cs_0.15_FA_0.85_PbI_2.85_Br_0.15_ PVK.

CH(NH_2_)_2_I (FAI) (99%, Sigma-Aldrich), CsBr (99.999%, Sigma-Aldrich), and PbI_2_ (99.999%, Thermal Scientific) precursors were used as received. Before sample preparation, the bare quartz sheet was cleaned with acetone and ethanol and then treated in UV plasma cleaning for 5 min. PVK films were fabricated using a simplified approach consisting of a single-cycle deposition method described elsewhere.^27^ The schematic illustration of the sequential thermal evaporation process is depicted in Figure 1a. During the deposition of the Cs_0.15_FA_0.85_PbI_2.85_Br_0.15_ film, three precursors were sequentially evaporated with the order of PbI_2_, FAI, and CsBr into one stack with a total thickness of 250 nm. Afterward, the second stack is deposited repeating the same sequenced three-layer precursors stack to reach a target thickness of 450 nm. The detailed precursor deposition parameters are listed in Table S1. Between the two deposition cycles, we applied different intermediate annealing (*T*_inter_) treatments on a hot plate for 5 min (without annealing (w/o-A); 50, 100, 130, and 160 °C) in a nitrogen-filled glovebox. The final PVK layer consisting of two stacks was annealed at 130 °C for 5 min, named *T*_final_, also in a N_2_-filled glovebox.

**Figure 1:**
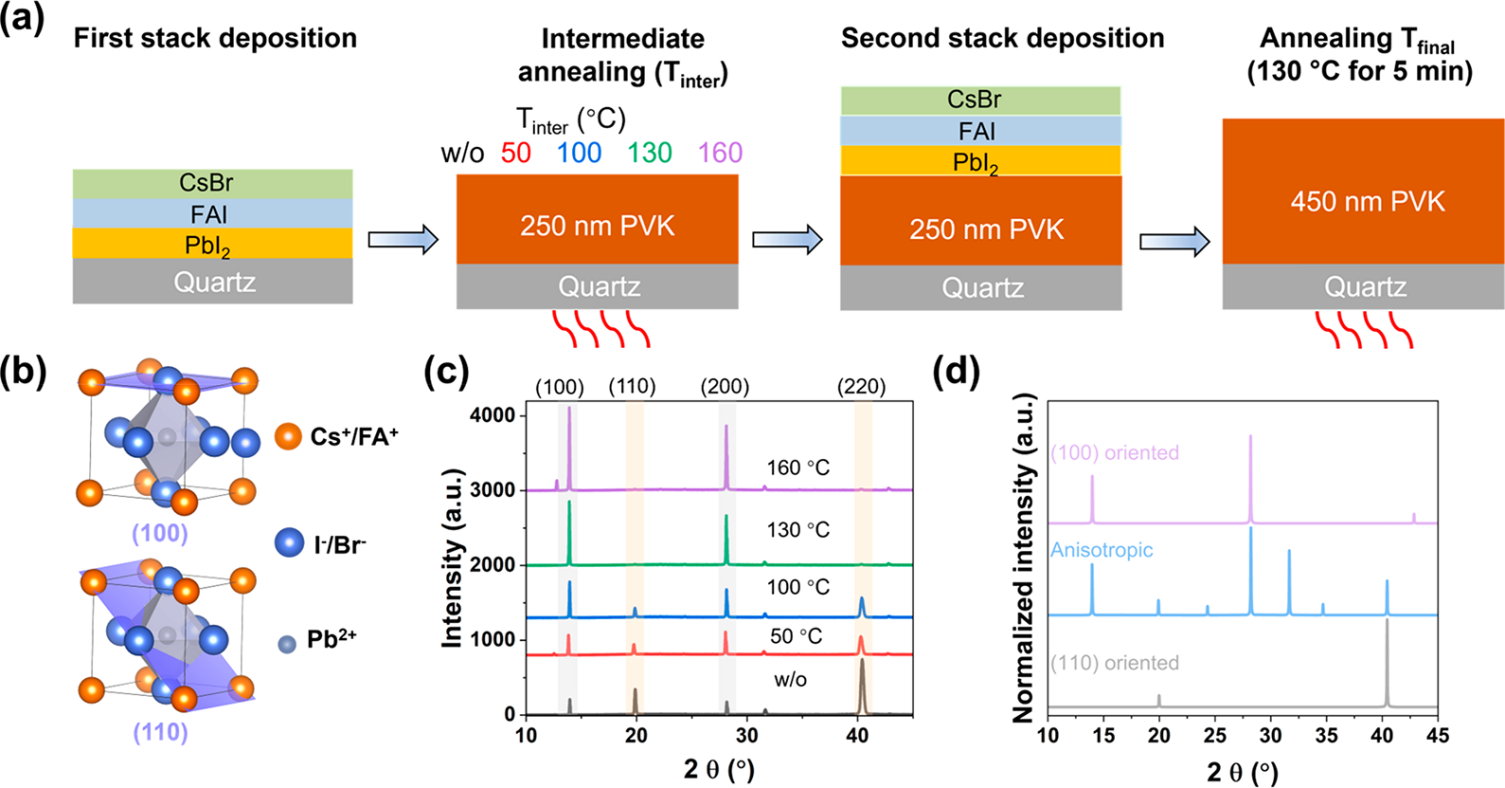
(a) Schematic illustration of the sequential thermal evaporation with various *T*_inter_. (b) Illustrations of the (100) and (110) planes in a unit cell. (c) XRD patterns of final PVK films grown with different *T*_inter_ values and constant final annealing (*T*_final_). (d) Simulated XRD patterns of nonoriented PVK and PVK with orientations along the (110) or (100) direction.

X-ray diffraction (XRD) patterns were measured with a Bruker D8 Advance diffractometer equipped with a Cu Kα X-ray source that has a wavelength of 1.54 Å.

Two-dimensional X-ray diffraction (2D-XRD, Bruker D8 Discover, Cu Kα) was performed with an incident angle of 5° to analyze the crystallinity and orientation within the PVK films. The X-ray generator shows a voltage of 40 kV and a current of 25 mA. The beam size is 2.0 mm in diameter. The intensity of the peaks is integrated with DIFFRAC.EVA software.

The elemental compositions of the PVK films were analyzed by X-ray photoelectron spectroscopy (XPS) using a ThermoScientific K-Alpha spectrometer. The spectrometer was equipped with a focused monochromatic Al-kα X-ray source (1486.6 eV) operating at 36 W (12 kV, 3 mA). The samples were transferred in a N_2_ box for XPS measurements but were exposed to air during sample loading. Peak fitting was performed with Avantage software using a Gaussian function. The binding energy was corrected for the charge shift by taking the primary C 1s hydrocarbon peak at BE = 284.8 eV as a reference.

The morphology of the PVK surfaces was measured by a scanning electron microscope (SEM, Thermo Scientific, Verios G4 UC) at an accelerating voltage of 5 kV with the secondary electron (SE) mode.

The absorptance of samples were measured by ultraviolet–visible spectroscopy (UV/vis, PerkinElmer, Lambda 950) with a wavelength range of 300–850 nm.

The photoluminescence spectra of the samples were investigated by steady-state photoluminescence (PL, HORIBA, FL3-111) with an excitation wavelength of 405 nm; besides, the emission light was filtered by a 550 nm filter.

Time-resolved microwave conductance (TRMC) was applied to learn about the carrier lifetime, mobility, and trap densities. All the measurement parameters can be found in ref (28). The effective electron and hole mobilities (∑μ) are derived from the maximum signal height (Δ*G*_max_), which is normalized by the absorptance at an excitation wavelength of 650 nm. The charge carrier half-lifetime is obtained from the photoconductance decay.

Kelvin probe force microscopy (KPFM, Bruker, Dimension Edge scanning probe microscope) measurements were performed in an enclosure provided by the manufacturer at ambient pressure, temperature, and humidity conditions. The requisite CPD distribution over the scan area (1 × 1 μm^2^) of the samples studied was determined by applying an AC voltage and plus a DC voltage to the AFM tip. To ensure the appropriate electrical conductivity between the tip and the sample, copper tape was attached between the film and the AFM holder. The contact potential difference (CPD) between the tip and sample was measured simultaneously with the topography of the region studied. The CPD/VPD distribution maps and topographical maps were collected with a similar pixel density of about 256 × 256 pixels per image and a scan rate of 0.6 μm/s. The microscope was equipped with an antimony (n) doped silicon tip (SCM-PIT-V2, Bruker) with a radius of 25 nm, coated on both front and back sides with platinum–iridium (Pt–Ir) material, to laterally resolve Volta potential difference distributions for PVK layers. The above tip was supported on an antimony (n) doped silicon cantilever, coated only on the backside with Pt–Ir material, possessing a resistivity in the range of 0.01–0.025 Ω/cm and a thickness of 2.8 μm. The data were analyzed with the Gwyddion software. To remove high-frequency noise, the KPFM figures were processed with a low pass filter.

After finishing both cycles and a final annealing step at *T*_final_ = 130 °C, we investigated the influence of the intermediate annealing temperature (*T*_inter_) in terms of crystallography. Figure 1b shows visual images of the unit cell with crystal orientation along the (100) and (110) planes. The XRD patterns of the final Cs_0.15_FA_0.85_PbI_2.85_Br_0.15_ films with different *T*_inter_ values are plotted in Figure 1c. The major peaks are located 2θ at 14.05°, 20.08°, 28.16°, 31.88°, and 40.43°, which are assigned to the (100), (110), (200), (012), and (220) crystal planes of PVK, respectively.^29^ Apparently, the (100) peak intensity (highlighted in light gray in Figure 1c) gradually increases with higher *T*_inter_. This trend is indicative of improved crystallization and crystal orientation of (100) direction for a higher thermal budget. On the contrary, by increasing the *T*_inter_ from w/o-A to 160 °C, the (110) peak intensity (highlighted in light brown) decreases to almost zero. To analyze the orientation of the prepared samples, simulated XRD patterns are provided in Figure 1d, which either are isotropic or have a preferred growth along the (110) or (100) direction. On comparison of the experimental patterns (Figure 1c) with the simulated results (Figure 1d), we note that intermediate annealing can control the crystal orientation ranging from (1) no annealing: some preferential orientation along the 110 direction; (2) *T*_inter_ = 100 °C annealing: near isotropic orientation; and (3) *T*_inter_ = 160 °C annealing: preferential orientation mostly along the (100) direction. The difference in full width at half-maximum (FWHM) of the (100) and (110) peaks is negligible as *T*_inter_ increased, as shown in Table S2, suggesting a comparable crystallite size in all samples. The top-view SEM images of these samples are shown in Figure S1. In agreement with the XRD results, there is no clear grain size variation as the crystal orientation changes. We show in Figure S2 the grain size distribution for samples with *T*_inter_ of w/o-A((110)/(100)-mixed) and 160 °C ((100)-oriented). Thus, an apparent preferable growth along the (100) and (200) crystallographic planes for PVKs by increasing the *T*_inter_ is concluded.

To further assess the controllable crystal orientations, we measured 2D-XRD of PVK samples w/o-A at 130 °C as shown in Figure 2. The two samples are selected based on their clear different preferential crystal orientations of mixed (110)/(100) and (100). In the 2D-XRD images of Figure 2a,b, the azimuthal intensity distribution correlates to the orientation of the planes.^30^ Therefore, uniform intensity along the Debye–Scherrer ring indicates no preferable crystal orientation.^31^ In contrast, the brighter parts compared with the dark region suggest specific out-of-plane orientations. The Debye–Scherrer ring shown in Figure 2a,b is the diffraction pattern of the (100) crystal plane for samples of w/o-A and 130 °C.^30^ It is observed that diffraction intensities of the preferential (100) plane varied significantly with different *T*_inter_, according to Figures 2a and 2b. To better visualize and compare the difference, we integrate the intensity of the (100) crystal plane along different azimuth angles (χ), as shown in Figures 2c and 2d. More details about the integration process can be found in Figure S3. The peak position reflects the angle of the (100) plane with respect to the substrate, while the peak intensity and width stand for the distribution.^32^ For sample w/o-A (Figure 2c), two broad peaks appear at 88–93° and 134–135° with relatively low peak intensities, suggesting that the (100) plane is oriented along two different directions with azimuth values of 88–93° and 134–135°, as visualized in the inset. In contrast, the sample with a *T*_inter_ of 130 °C (Figure 2d) shows an increased peak intensity with a narrower distribution of the (100) plane oriented along 89°–90°, as shown by the cubes in the inset. Apparently, as *T*_inter_ increases, the orientation of the (100) plane is intensified along χ ≈ 90°, which is perpendicular to the substrate surface. It confirms that the preferred facet orientation along the (100) crystal plane is achieved by applying a high *T*_inter_.

**Figure 2:**
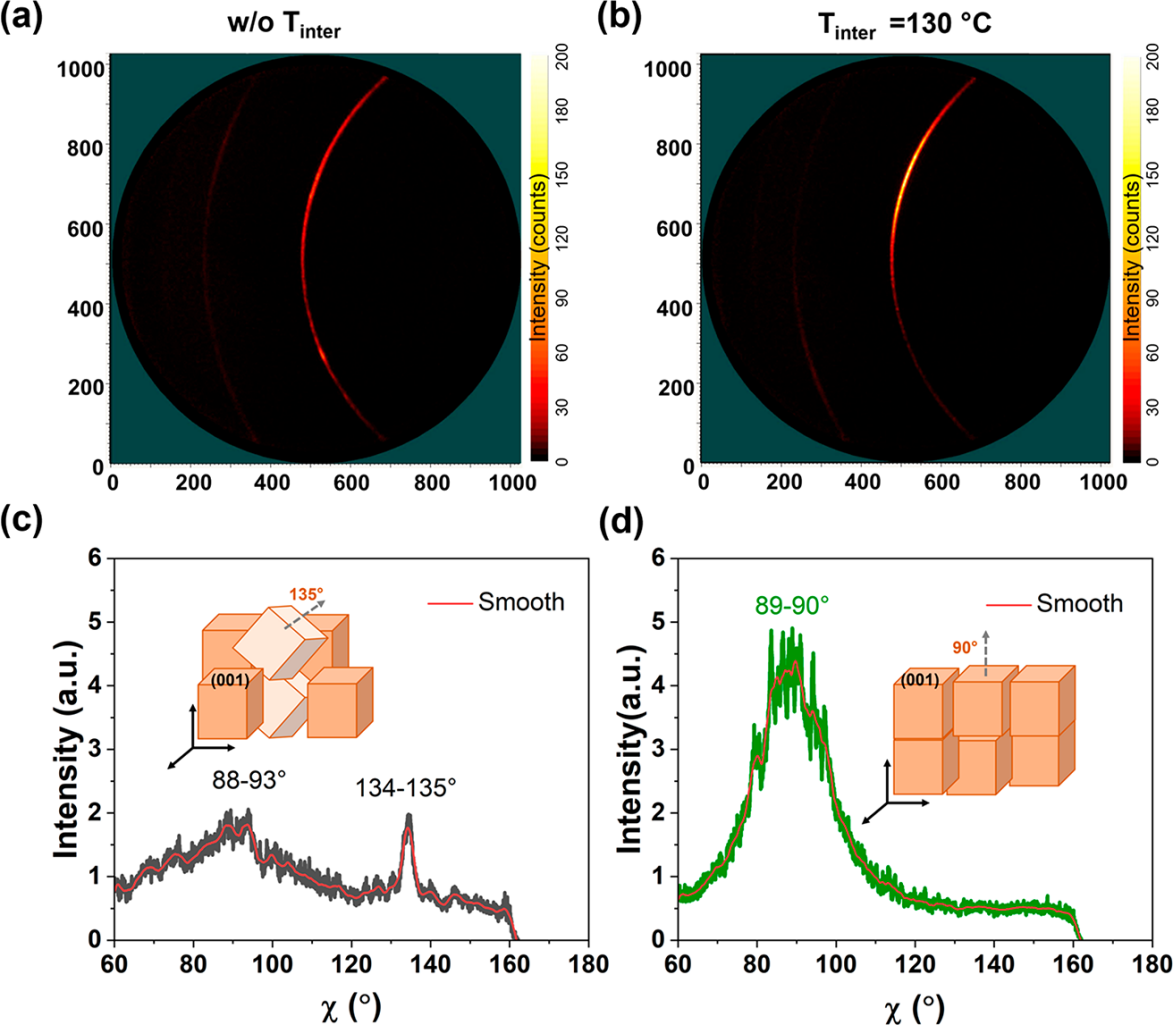
(a, b) 2D-XRD of samples with *T*_inter_ values of (a) w/o-A and (b) 130 °C. (c, d) Integration of the azimuthal intensity along the (100) reflex in 2D-XRD for samples with *T*_inter_ of (a) w/o-A and (b) 130 °C. The insets in (c) and (d) depict the preferred crystal orientation.

To analyze the influence of the stack from the first cycle on PVK growth, XRD characterization is performed on the first stack after the different annealing temperatures, as shown in Figure 3a. For the samples w/o-A and *T*_inter_ = 50 °C, XRD patterns show a high PbI_2_ peak at 2θ = 12.7°, indicating a low conversion of the deposited precursors into PVK. With the increase of *T*_inter_ to 100 °C and higher, the PbI_2_ peak disappears, and the PVK peak gradually increases in intensity. Then we deposited the second stack on these various samples and applied a final annealing step for all samples at 130 °C. The diffraction patterns of these double-cycle deposited PVKs are shown in Figure 3b. The (110) and (220) plane diffraction signals in Figure 3b gradually disappear as *T*_inter_ increases, while the (100) and (200) PVK diffraction peaks become more intense. The *I*_PVK_ (sum of peak intensities of (100) and (110)) of the first stack and the (100)/(110) ratios of the PVK films are plotted in Figure 3c as a function of *T*_inter_. For *T*_inter_ < 100 °C the *I*_PVK_ vs *T*_inter_ is nearly constant. When *T*_inter_ is around 100 °C, the ratio quickly increases, confirming the nearly complete PVK formation. A *T*_inter_ of 100 °C seems to be a threshold temperature to obtain substantial precursor conversion which is in line with other works on vacuum deposited PVKs.^33−35^ Interestingly, the trend for the (100)/(110) ratio for the final PVK with increasing *T*_inter_ follows the same trend as the *I*_PVK_ obtained from the first stack. For low *T*_inter_, the (100)/(110) ratio remains below 1, while for a high *T*_inter_, a (100)/(110) ratio >10 is found, demonstrating a substantial change in the preferred PVK orientation. To analyze the preferential orientation of the grown PVK layers with different values of *T*_inter_, the peak intensities for layers with different orientations were simulated and can be found in Figure S4 and Table S3. These X-ray diffraction patterns have increasing preferential ordering along the (100) or (110) directions as defined by the March–Dollase parameter (MDP)^36^ with a MDP of 1 corresponding to the absolute random orientation and a lower MDP value corresponding to increasing preferential orientation.^36^ The sample with *T*_inter_ = 100 °C shows peak intensities comparable to those of the simulation with no preferential oriented PVK (MDP = 1). From our analysis, we can conclude that the degree of preferential PVK growth is in line with the conversion of the precursors in the first stack. We ascribe the crystal orientation dependency on the *T*_inter_ to a template-guided PVK growth process. In other words, the ratio of unreacted precursors/converted PVK at the surface of the first stack dictates the preferential ordering of the second stack PVK. This finally leads to the manipulation of PVK orientation from a mixed (100) and (110) to a preferential (100).

**Figure 3:**
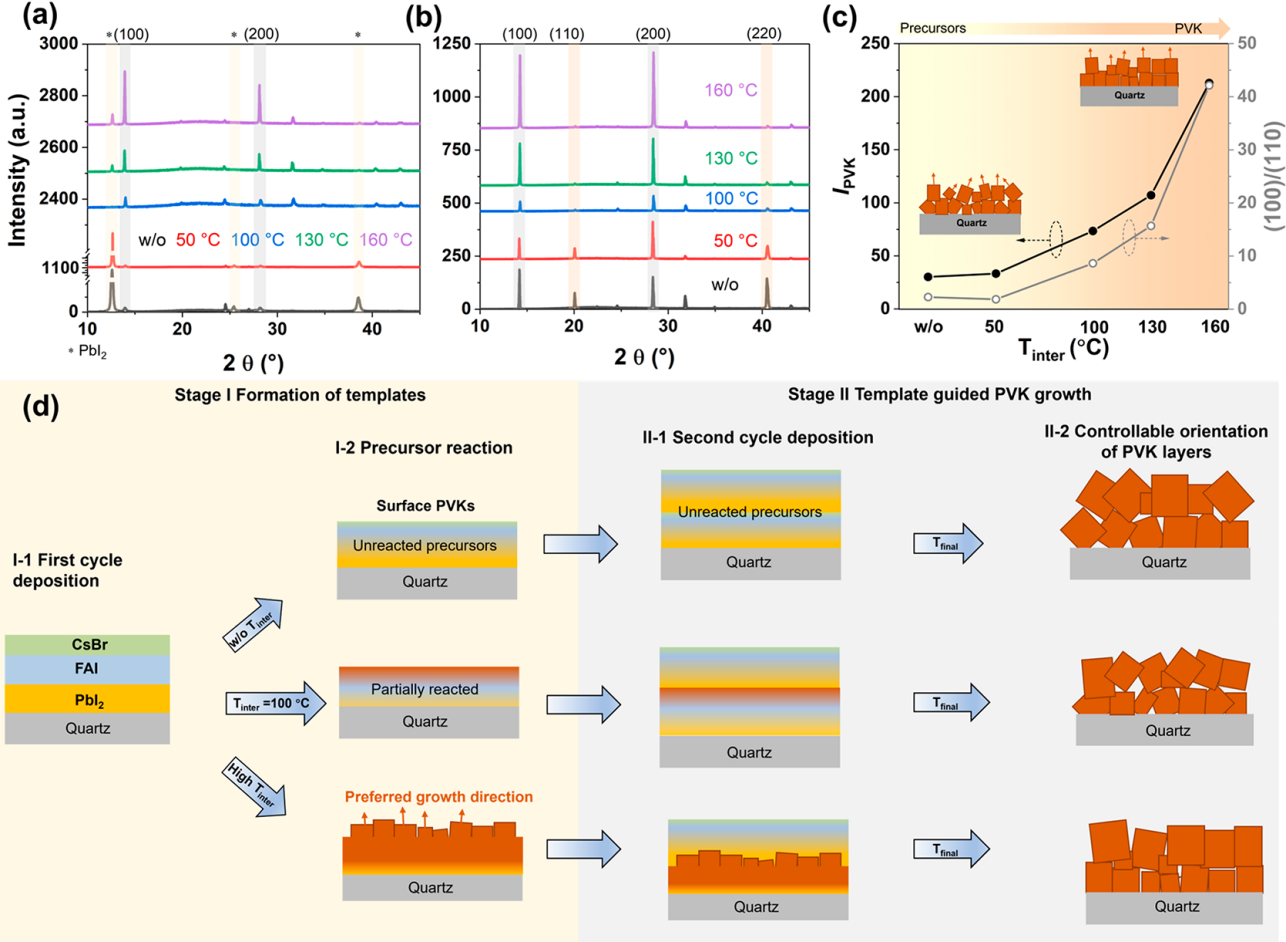
(a) XRD patterns of the first stack PVKs with different *T*_inter_. Note that the *y*-axis contains two gaps to improve clarity. (b) XRD patterns of the double-cycle deposited samples which are continued with the first-cycle deposited samples shown in Figure 3a. (c) PVK peak intensity (*I*_PVK,_ which is the sum of intensities of peak (100) and (110)) of the first stack and the (100)/(110) ratio of the corresponding double-cycle deposited films as a function of *T*_inter_. (d) Mechanism of template-guided PVK crystal orientation growth for different *T*_inter_.

To further confirm this explanation, XPS is applied to the first stacks w/o-A and with *T*_inter_ = 160 °C. Full XPS spectra for the first stack samples w/o-A and 160 °C can be found in Figure S5, while the high-resolution spectra of Pb 4f_7/2_, I 3d_5/2_, Cs 3d_5/2_, and Br 3d are shown in Figure S6. The corresponding peak positions and atomic percentages are given in Tables S4 and S5. The peak position of Pb 4f_7/2_ shows 138.5 eV for sample w/o-A, while it shifts to 139.1 eV for the sample with 160 °C (Table S4). These peak positions are all calibrated by C 1s spectra at 284.8 eV.^37^ Interestingly, the reported binding energy of Pb 4f_7/2_ in PbI_2_ for the literature value is 138.5 eV,^37^ indicating that the Pb still exists in the form of PbI_2_ on the surface of the sample w/o-A. To support this conclusion, we also recorded high-resolution XPS spectra of Pb 4f_7/2_, I 3d_5/2_, Cs 3d_5/2_, and Br 3d of the individual precursors shown in Figure S7 and Table S6. Furthermore, the ratio of FAI/PbI_2_ at the film surface can be obtained by calculating the (I-2Pb)/Pb ratio, which is 1.71 and 0.91 for the samples w/o-A and *T*_inter_ = 160 °C, respectively (Table S5). Apparently, the difference in (I-2Pb)/Pb for these two samples provides different templates for the growth of the next stack. Hence, the degree of PVK formation of the first stack directly influences the growth and crystal orientation of the entire PVK film.

Based on the XRD, 2D-XRD, and XPS results, we relate the crystal orientation dependency on the *T*_inter_ to a template-guided PVK growth process, as shown in Figure 3d. Different intermediate stages are achieved using *T*_inter_ from w/o-A to 160 °C. For the case without *T*_inter_, the unreacted precursors in the film provide a disordered template for the deposition of the second stack and lead to a PVK film with mixed orientations as shown. On the contrary, the samples with high *T*_inter_ combined with nearly complete PVK conversion provide a template for the deposition of the second stack leading to a PVK film with a highly preferential orientation along the (100) direction.

As reported in several publications,^22,38,39^ solution-based PVKs show optoelectronic properties dependent on crystal orientation. Hereby, we investigate the influence of orientation growth on optoelectronic properties based on thermally evaporated PVKs. Specifically, we choose samples with a *T*_inter_ of w/o-A, *T*_inter_ = 100 °C, and *T*_inter_ = 160 °C to study the optoelectronic properties. UV–vis and PL results of the first stacks with different *T*_inter_ values are shown in Figure 4a–c. In Figure 4a. The first stack w/o-A shows relatively low absorptance compared to the other samples because of the limited PVK conversion, as confirmed by XRD in Figure 3a. Both steady-state PL and time-resolved PL measurements are performed to study the radiative band-to-band recombination at an excitation wavelength of 405 nm. The appearance of two peaks for samples w/o-A in Figure 4b could be explained from the initial formation of different PVK compositions^31^ or trap-assisted emission.^40^ Because the (100) PVK peak in the XRD pattern can be fitted using a single Gaussian as shown in Figure S8, it is expected that the PVK composition is rather uniform. This is in line with the very similar absorption onsets shown in Figure 4a for different values of *T*_inter_. Hence, it is likely that the presence of unreacted precursors might lead to the formation of shallow emissive states,^41^ resulting in the appearance of the shoulder in the PL spectrum at 810 nm. The position of maximum PL in Figure 4b shows a gradual blue-shift with increasing *T*_inter_ as evidence of the precursor reaction, which is consistent with the conclusion from Figure 3. Similarly, the longer lifetime in TRPL decay of the first stack with 160 °C shows a more complete conversion. The first stacks treated with different T_inter_ (Figures 4a–c) are then completed with the second cycle and final annealing step as shown in Figures 4d–f. The onset of absorptance in Figure 4d is identical for all three samples, implying that the bandgap is identical for the PVKs with different preferential orientation. From the increased PL with higher values for *T*_inter_, the radiative electron–hole recombination increases as shown in Figure 4e. In addition, the TRPL measurements shown in Figure 4f exhibit a slower decay over time with increasing *T*_inter_. All the TRPL spectra are fitted by monoexponential decays, and the corresponding lifetimes and deviation (χ^2^) are summarized in Table S7. These PL measurements indicate that at least the surface of the PVKs prepared using different values for *T*_inter_ develops different surface properties.

**Figure 4:**
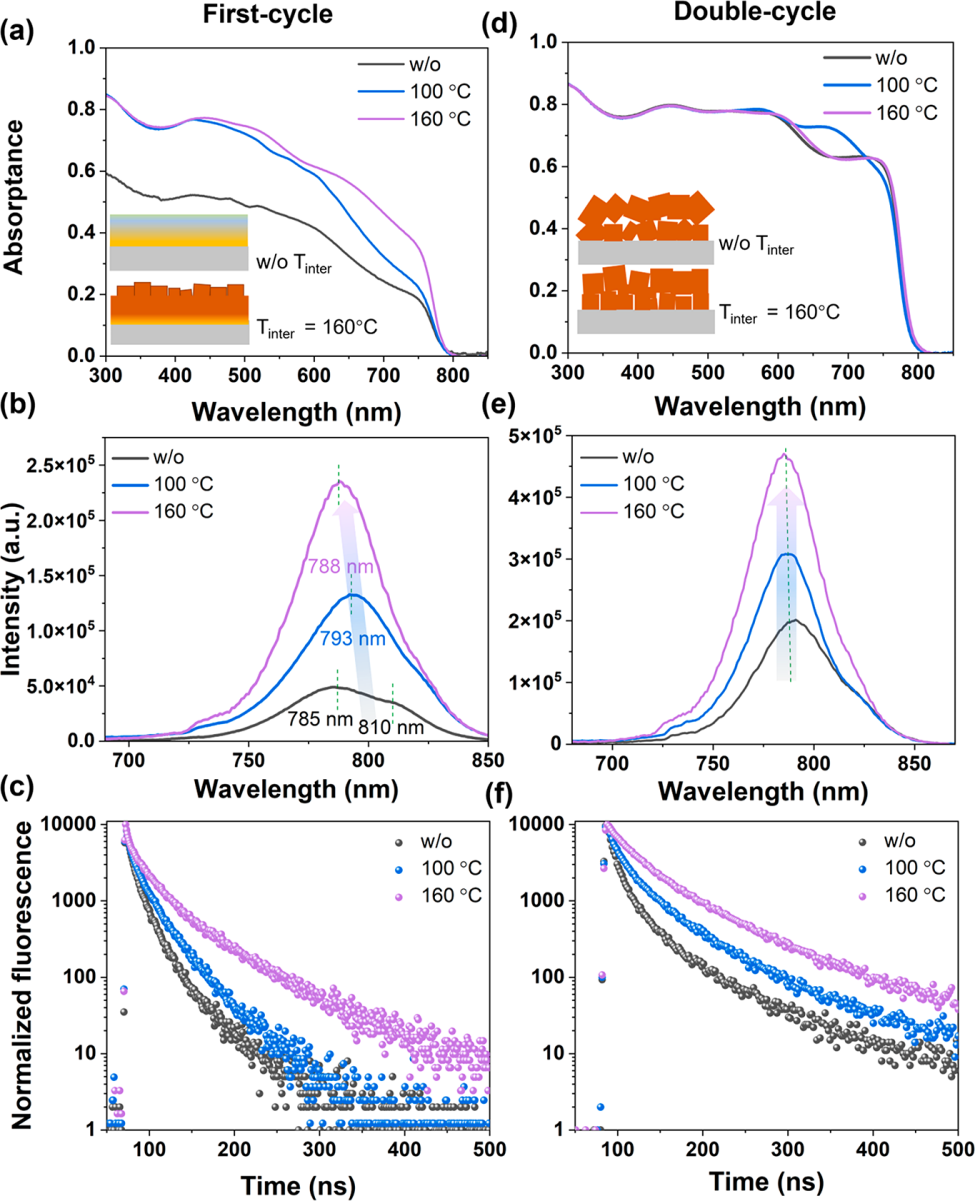
Optoelectronic properties of the first stack (left column) and double-stacked (right column) samples with different *T*_inter_: (a, d) absorbance spectra, (b, e) steady-state PL, and (c, f) TRPL.

TRMC gives information regarding charge carrier mobilities and lifetimes. Figures S9a–c show the photoconductance (Δ*G*) as a function of time on pulsed excitation for the samples w/o-A, *T*_inter_ = 100 °C, and *T*_inter_ = 160 °C. Given the low exciton binding energy of CsFA-based PVKs, we can assume that the charge carrier generation yield, φ, is close to unity at room temperature.^42^ No significant differences in both the lifetime and mobility are observed between samples with different orientations, as summarized in Figure 5a. The recombination dynamics are also investigated by probing the sample with different laser intensities, as shown in Figures S9a–c. The decay of Δ*G* under low photon intensities (10^9^ /cm^2^) is attributed to the immobilization of excess charge carriers via trapping or recombination,^28^ indicated by process 1 in Figure S9d. As the photon intensity increases to 10^10^ /cm^2^, the decay curve shows a clear difference with the low photon intensity for all samples (Figures S9a–c), demonstrating enhanced second-order electron–hole recombination, as shown by process 2 in Figure S9d. Therefore, we conclude that all samples with different preferable crystal growth share a comparable trap density. We find similar mobility values of 30 cm^2^/(V s) and τ_1/2_ values of 780 ns using photon intensities of 10^10^/cm^2^ for all samples, as summarized in Figure 5a. In view of the excitation wavelength used for the TRMC measurements (650 nm), resulting in a rather homogeneous excitation profile, we conclude that the bulk properties of the different PVKs are very similar. This is agreement with the comparable grain size for the different samples, as verified by both SEM (Figures S1 and S2) and XRD (Table S2). Therefore, the comparable charge carrier mobilities and lifetimes of samples with different values for *T*_inter_ imply that the bulk optoelectronic properties for thermally evaporated PVKs crystals are crystal orientation independent.

**Figure 5:**
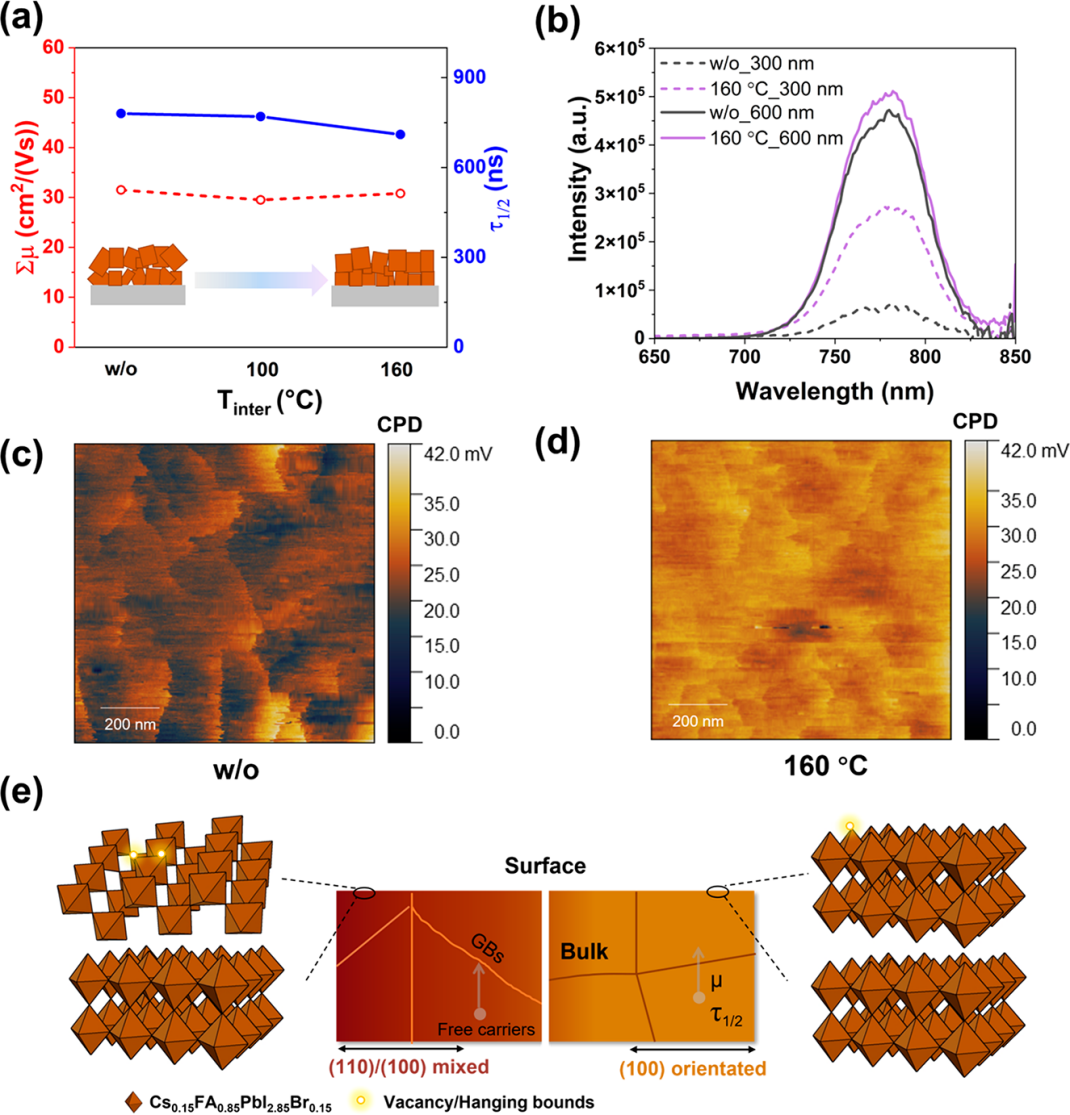
(a) Mobility (left axis) and half-lifetime (right axis) extracted from TRMC traces in Figure S6 as a function of *T*_inter_ for different samples. (b) PL spectra of samples w/o A and *T*_inter_ = 160 °C with excitation wavelengths of 300 and 600 nm. (c, d) 2D CPD distribution maps of double-stacked PVK films with *T*_inter_ of (c) w/o-A, and (d) 160 °C. (e) Schematic illustration of the relationship between crystal orientation and the corresponding bulk/surface optoelectrical properties.

According to DFT calculations reported by Li et al.,^23^ the (100) planes show less dangling bonds as compared to the (110) planes in accordance with our observations. In this work, the reduced standard deviation for the sample with *T*_inter_ = 160 °C agrees with the fact that at the surface, only a single plane is exposed. For the sample w/o-A the different planes lead to larger variations in measured CPD values.

In order to examine how the extent of preferential ordering affects the optoelectronic properties, PL spectra are recorded using different excitation wavelengths (λ = 300 nm or λ = 600 nm) of the samples w/o-A and with *T*_inter_ = 160 °C, as shown in Figure 5b. The penetration depths are very different going from tens of nanometers to hundreds of nanometers. While the PL spectra are similar for both samples using λ = 600 nm, at λ = 300 nm the intensity for the sample w/o A is a factor of 4 lower than that of the sample with *T*_inter_ = 160 °C. This implies that the surface properties of the sample w/o A lead to more radiationless decay. To further confirm our conclusion, we performed additional Kelvin probe force microscopy (KPFM) measurements to specifically study the laterally resolved contact potential difference distribution. Figures 5c and 5d show KPFM images of the contact potential difference (CPD) distribution for the samples w/o-A and T_inter_ = 160 °C, respectively. The sample w/o-A exhibits a low averaged CPD of 22.8 mV combined with a large standard deviation of ±0.95 mV. On the contrary, the sample with *T*_inter_ = 160 °C shown in Figure 5d shows a large and quite uniform averaged CPD of 29.7 mV and a standard deviation of ±0.47 mV. The CPD distributions of both images are converted into histograms in Figure S10. The increased average CPD is related to a reduced concentration of surface defects caused by the dangling bonds.^43^ According to DFT calculations reported by Li et al.,^23^ the (100) planes show less dangling bonds as compared to the (110) planes in accordance with our observations. In this work, the reduced standard deviation for the sample with *T*_inter_ = 160 °C agrees with the fact that at the surface only a single plane is exposed. For the sample with w/o-A the different planes lead to larger variations in measured CPD values.

Combining the results of measurements probing the bulk and surface, the influence of thermal evaporated PVK crystal orientation on film optoelectrical properties is schematically illustrated in Figure 5e. In terms of bulk properties, variation in the preferable crystal growth has little effect on the bulk optoelectronic properties. Reasons lay in the similar grain boundaries, as confirmed by the comparable grain size in Figure S2. On the contrary, the effect of different crystal orientations on the optoelectronic properties is observed at the surface of the film. The mixed crystal orientations expose different facets at the film surface, affecting the number of surface vacancies and dangling bonds and further exhibiting differences in surface potential. A uniform ordering of the PVK crystals might lead to improved charge extraction at the interface of PVK and transporting layers and less interfacial recombination.^44^

We propose an intermediate annealing approach to manipulate the crystal orientation of thermally evaporated Cs_0.15_FA_0.85_PbI_2.85_Br_0.15_ PVK films fabricated by using a simplified sequential layer deposition method. By optimizing the intermediate annealing temperature, we demonstrate that the crystal orientation can be tailored ranging from primarily (110) to near isotropic to predominantly (100). Our results indicate that the degree of precursor reaction upon different intermediate annealing temperatures of the first stack influences the preferred crystal orientation of the entire PVK film by providing different templates for the subsequent deposition. Moreover, we reveal how thermally evaporated PVK crystal orientation influences the film optoelectronic properties by applying PL, TRMC, and KPFM measurements. We found that the preferable growth along both directions have comparable bulk properties in terms of charge carrier mobility, lifetime, and trap densities, which is dominated by the grain size and grain boundaries. On the contrary, the (100) oriented growth exhibits higher surface potential/lower trap densities at the film surface compared to the primarily (110) to near isotropic PVK layers, which plays a decisive role in charge carrier extraction. Therefore, realizing uniform PVK layers with a highly preferential (100) orientation is expected to show improved charge extraction and transportation at the device level.

## Supporting Information Available

Top-view SEM images, grain size distribution, integration of the 2D XRD in software, simulated XRD patterns, XPS spectra, XRD + fits, detailed TRMC results, a table for thermal evaporation (temperature, deposition rate, and vacuum) (PDF)
