# Supplementary material for: Vacuum Deposited Perovskites with a Controllable Crystal Orientation
Source: J Phys Chem Lett. 2023 Sep 25;14(39):8787–95. doi: 10.1021/acs.jpclett.3c01920 (PMC10561267; doi:10.1021/acs.jpclett.3c01920)
Supplement: Supplementary file 1 — jz3c01920_si_001.pdf [file jz3c01920_si_001.pdf]

Supporting Information

Vacuum Deposited Perovskites with a Controllable Crystal Orientation

Jin Yan[1,2], Lena Sophie Stickel[1,3], Lennart van den Hengel[2], Haoxu Wang[1], Prasaanth Ravi Anusuyadevi[4], Agnieszka Kooijman[4], Xiaohui Liu[2], Bahiya Ibrahim[2], Arjan Mol[4], Peyman Taheri[4], Luana Mazzarella[1], Olindo Isabella[1], Tom J. Savenije[2].*

1 PVMD group, Delft University of Technology, Mekelweg 4, 2628 CD Delft, the Netherlands

2 Department of ChemE, Delft University of Technology, Van der Maasweg 9, 2629 HZ Delft, the Netherlands

3 Georg-August-University Göttingen, Göttingen, 37077, Germany.

4 Department of Materials Science and Engineering, Delft University of Technology, 2628 CD Delft, the Netherlands

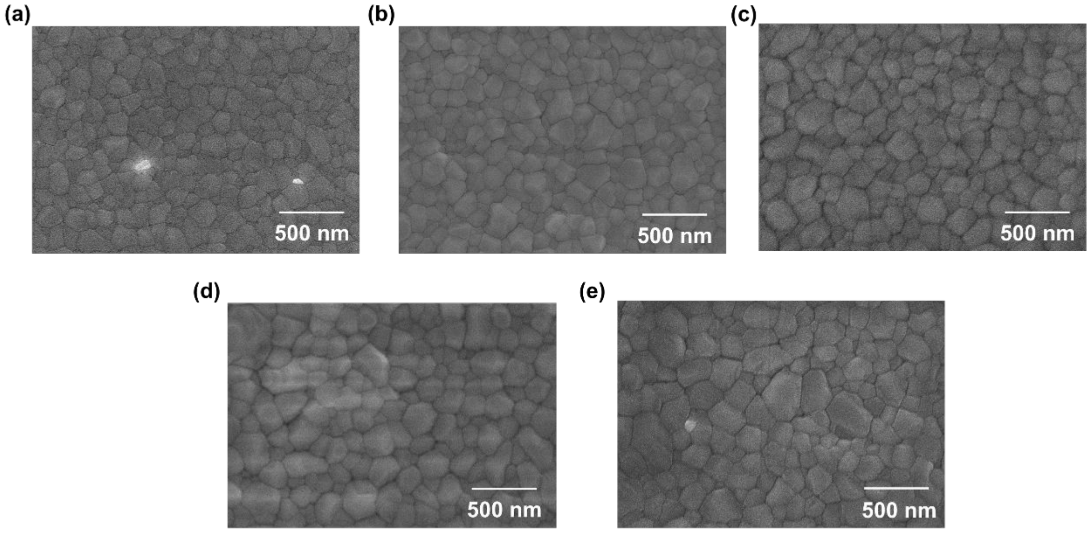

Figure S1 Top-view SEM of $Cs_{0.15}FA_{0.85}PbI_{2.85}Br_{0.15}$ samples with different T_{inter} **(a)** w/o-A, **(b)** 50 °C, **(c)** 100 °C, **(d)** 130 °C and **(e)** 160 °C.

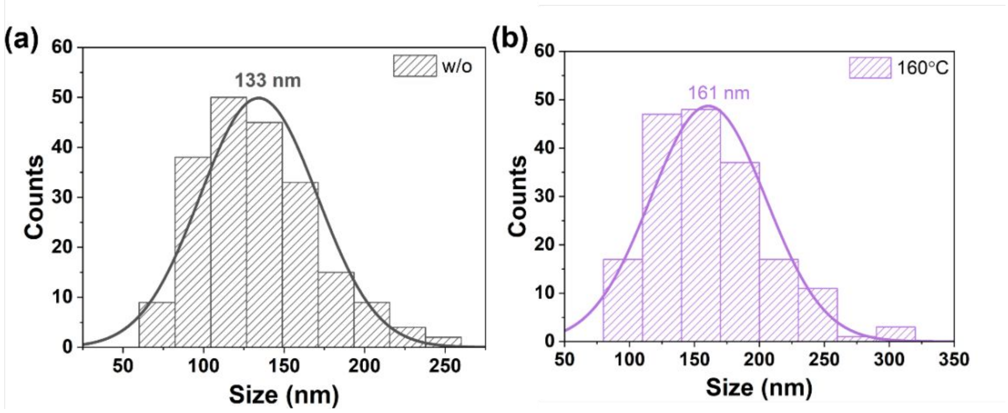

Figure S2 Grain size distribution of $Cs_{0.15}FA_{0.85}PbI_{2.85}Br_{0.15}$ samples **(a)** w/o-A and **(b)** T_{inter} of 160 °C reported in figure S1. The grain sizes are collected by using ImageJ software.

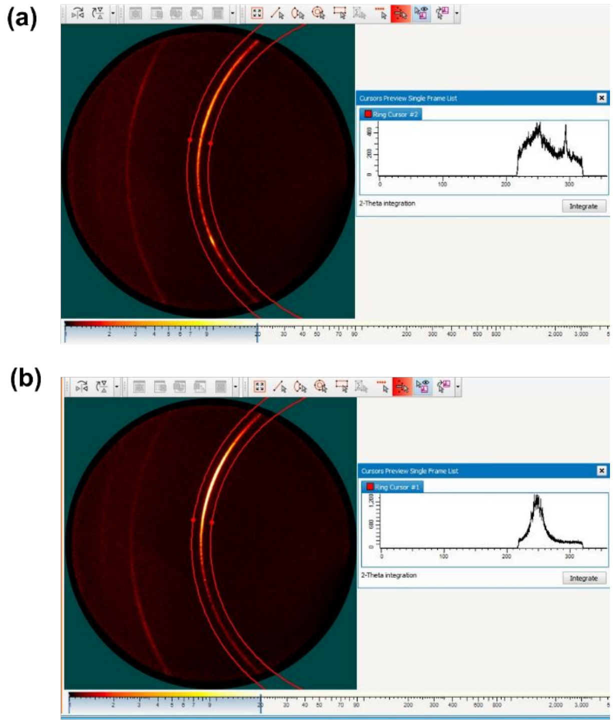

Figure S3 Integration of the (100) diffraction ring of 2D XRD pattern with software (DIFFRAC.EVA) **(a)** w/o-A **(b)** 130 °C.

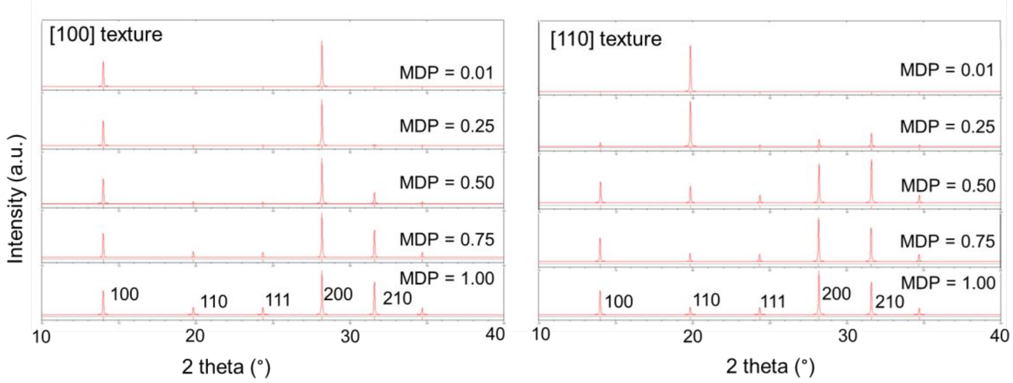

Figure S4 XRD pattern simulation of samples textured along (100) and (110) orientation with varying degrees of texture defined by the March–Dollase parameter (MDP). Software named PowderCell2.4 was used.

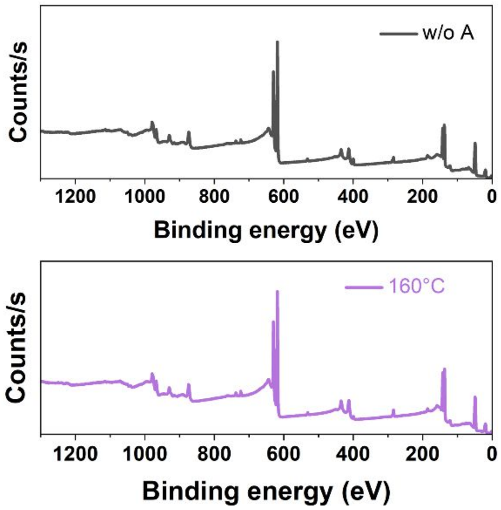

Figure S5 Wide XPS image of the samples with T_{inter} of w/o-A and 160 °C.

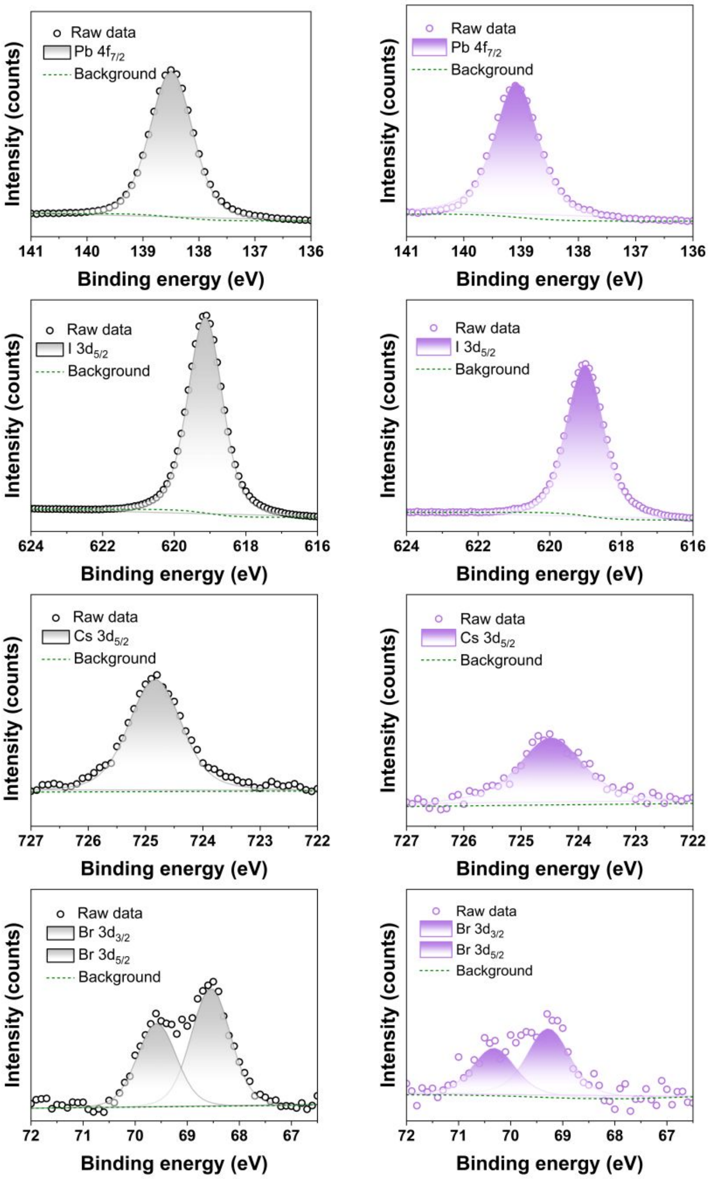

Figure S6 Fine XPS images of Pb $4f_{7/2}$, I $3d_{5/2}$, Cs $3d_{5/2}$, Br $3d_{5/2}$, and Br $3d_{3/2}$ peaks with different T_{inter} for first stacks.

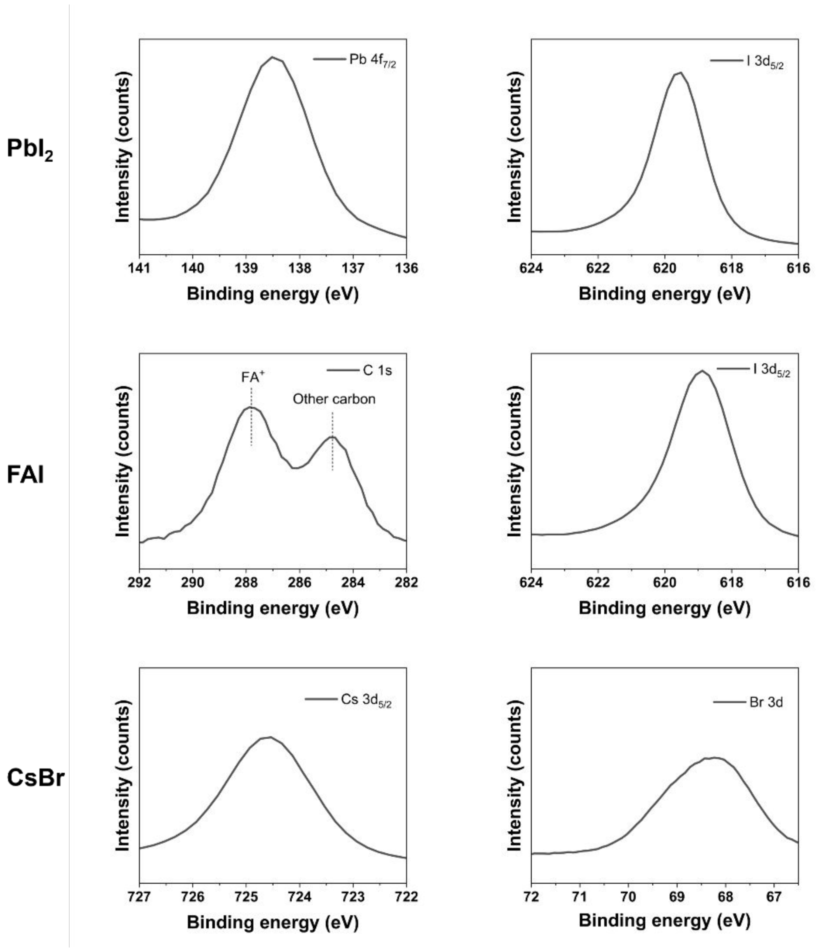

Figure S7 XPS spectrum of the pure precursor (PbI_2, FAI, and CsBr) with surface scan.

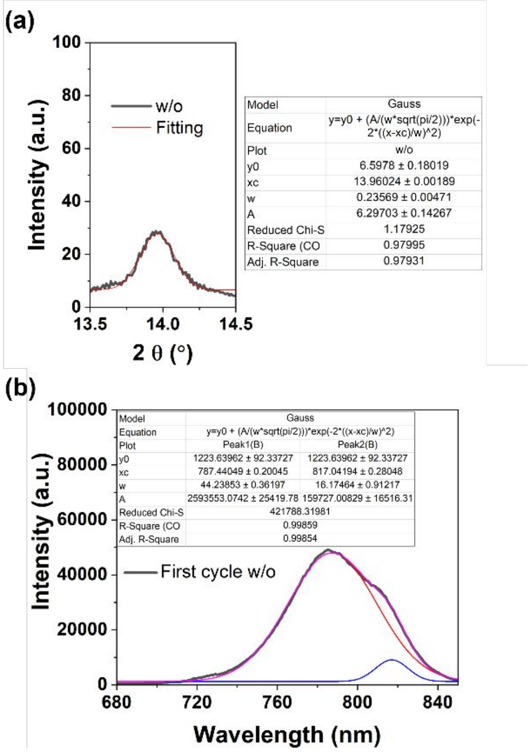

Figure S8 Gaussian fitting of **(a)** XRD pattern and **(b)** PL spectrum of the fist cycle-deposited sample w/o-A.

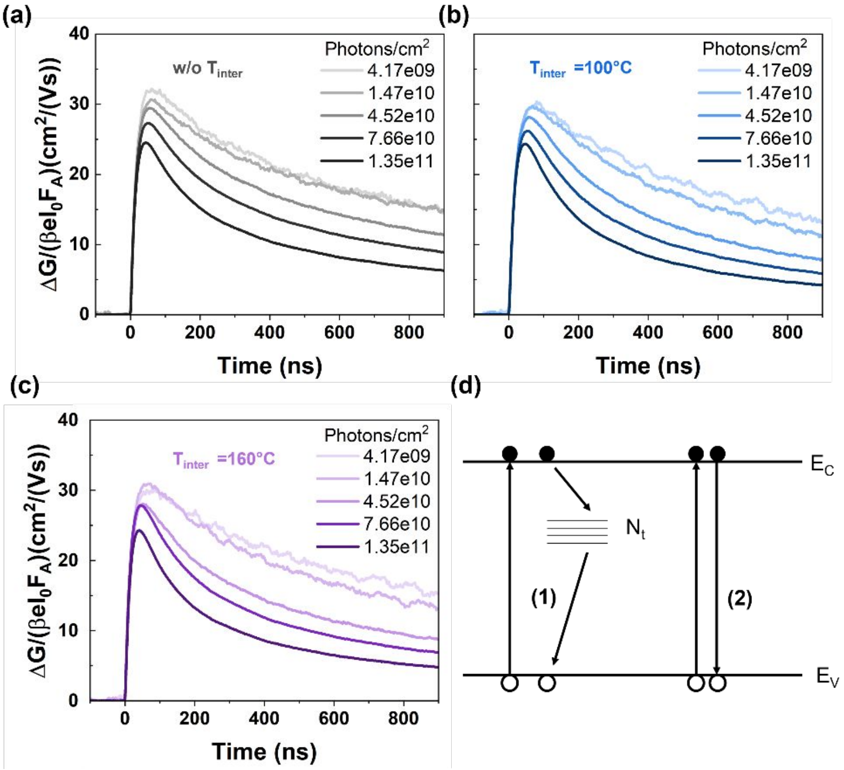

Figure S9 TRMC of samples measured under different photon intensities for different T_{inter}: **(a)** w/o-A, **(b)** 100 °C, and **(c)** 160 °C. **(d)** Kinetic model of the charge-carrier processes.

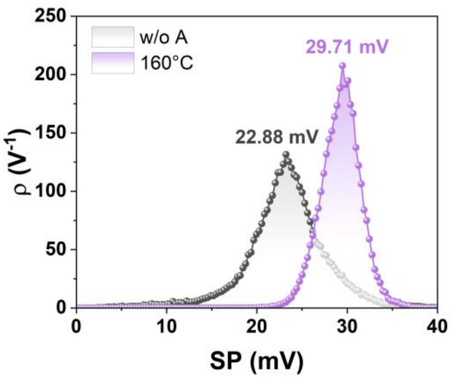

Figure S10 Histogram of the contact potential difference (CPD) distribution for PVKs with (100) preferable and (110)/(100) mixed orientation.

Table S1 Processing conditions for different precursors.

**Samples**	**Precursors**		
	**PbI_2**	**FAI**	**CsBr**
**Temperature (°C)**	202-236	72-108	387-391
**Rate (Å/s)**	0.4-0.5	0.4-0.5	0.08-0.10

**Vacuum (mbar)**	10^{-7}	10^{-6}	10^{-6}

Table S2 Full-width half-maximum (FWHM) of (100) and (110) peaks for samples with different T_{inter}.

T_{inter} (°C)	FWHM (100)	FWHM (110)
**w/o A**	0.130	0.170
**50**	0.148	0.191
**100**	0.154	0.191
**130**	0.148	-
**160**	0.145	-

Table S3 Simulated peak intensity with different preferred orientation/MDP value.

2 theta (°)	*hkl*	Intensity (a.u.) with [100] texture				
		MDP 1.00	MDP 0.75	MDP 0.50	MDP 0.25	MDP 0.01
13.99	100	100.00	100.00	100.00	100.00	100.00
19.83	110	30.72	23.58	7.16	0.39	0.00
24.35	111	29.41	21.53	6.01	0.31	0.00
**2 theta (°)**	***hkl***	**Intensity (a.u.) with [110] texture**				
		MDP 1.00	MDP 0.75	MDP 0.50	MDP 0.25	MDP 0.01
13.99	100	100.00	100.00	100.00	8.18	0.00
19.83	110	30.72	34.38	78.73	100.00	100.00
24.35	111	29.41	31.29	36.26	3.30	0.00

Table S4 Peak positions of cores in XPS spectra for samples with T_{inter} of w/o-A and 160 °C.

XPS	w/o A	160 °C	Ref [1]
**Pb $4f_{7/2}$**	138.5	139.1	138
**I $3d_{5/2}$**	619.1	619.0	619
**Cs $3d_{5/2}$**	724.8	724.5	724
**Br $3d_{5/2}$**	68.5	69.3	69

Table S5 Atomic percentage of Pb, I, Cs, and Br for samples with T_{inter} of w/o-A and 160 °C.

Atomic percentage	w/o A	160 °C
**Pb**	19.43	24.18
**I**	72.08	70.54
**Cs**	3.23	2.91
**Br**	5.26	2.37

Table S6 Peak positions in the XPS spectra of the precursors combined with the atomic ratio (data extracted from figure S7). Note that the information regarding C 1s is based on the FA^+ peak shown in figure S7.

Elements in pure precursors		Peak position	Atomic ratio
**PbI_2**	**Pb $4f_{7/2}$**	138.5	Pb/I = 0.51
	**I $3d_{5/2}$**	619.3	
**FAI**	**C 1s**	287.8	C/I = 1.02
	**I $3d_{5/2}$**	618.9	
**CsBr**	**Cs $3d_{5/2}$**	724.6	Cs/Br = 0.96
	**Br $3d_{5/2}$**	68.3	

Table S7 Decay times obtained from TRPL and fitted by monoexponential decay equation.

Samples	$\tau_{1/2}$ (ns)	χ^2
w/o A	45.98	0.998
100 °C	59.85	0.999
160 °C	83.14	0.999

Reference

[1] XPS Database

https://link.zhihu.com/?target=https%3A//link.jianshu.com/%3Ft%3Dhttps%3A//srdata.nist.gov/xps/selEnergyType.aspx
